# Associations of Depression Score with Dialkyl Phosphate Metabolites in Urine: A Cross-Sectional Study

**DOI:** 10.3390/brainsci14121290

**Published:** 2024-12-21

**Authors:** Hai Zhao, Xuejun Kang

**Affiliations:** Key Laboratory of Child Development and Learning Science (Ministry of Education), School of Biological Science and Medical Engineering, Southeast University, Nanjing 211189, China; 230218247@seu.edu.cn

**Keywords:** depression score, depression severity, PHQ-9, urinary dialkyl phosphate metabolites, NHANES

## Abstract

Objectives: Growing evidence suggests a link between organophosphate insecticides and depression disorder. These chemicals are metabolized and subsequently expelled through the urinary tract. The present study aims to investigate whether dialkyl phosphate metabolites associate with depression score and severity among the general population. Methods: This cross-sectional study used data from the National Health and Nutrition Examination Survey (NHANES). Depression was evaluated by the Patient Health Questionnaire-9 (PHQ-9). All urinary dialkyl phosphate metabolites were quantitatively analyzed. The survey’s complex design parameters and sampling weights were considered. Results: 3035 eligible individuals were included. The estimated prevalence of mild and major depression was 18.3% (95% confidence interval [CI]: 16.9–19.7%) and 9.9% (95% CI: 8.7–11.0%). For each incremental unit in the level of urinary dimethyl phosphate (DMP), individuals were found to have a higher depression score of 0.77 and a significantly increased odds ratio (OR) of 1.13 (95% CI: 1.12–1.13) for mild depression and 2.75 (95% CI: 2.74–2.76) for major depression. Conclusions: Our findings indicate positive and independent associations between urinary dialkyl phosphate metabolites and an elevated risk of depression among the general population.

## 1. Introduction

The WHO has compiled estimates of the prevalence of depression in all regions of the world for 2015 according to available data sources [1]. These calculations indicate that one-year prevalence of depression among females is slightly higher in Africa and the Americas (5.8%), and slightly lower in the Western Pacific region (4.2%) compared to other regions of the world (5.0%). The Institute for Health Metrics and Evaluation has created recent estimations of depression point prevalence in different worldwide areas as part of their Global Burden of Disease Study 2019 [2]. The point estimated value of prevalence among people was highest in North America (4.4% for women and 2.5% for men), but lowest in the Western Pacific (2.3% for women and 1.3% for men); the value was moderate for both women (2.8–3.6%) and men (1.9–2.0%) in the rest of the world. Depression was estimated to be the 4th, 6th, and 14th leading cause of global disability-adjusted life-years (DALYs) among people aged 10–24, 25–49, and 50–74 years, respectively, which brings a substantial health and economic burden to all societies [3]. What reasons account for such variations remains unclear. They might be attributed to the risk or protective factors. For instance, different reactions to questionnaires evaluating depressive symptoms might also explain some of the changes. Complex relationships of genetic, neurobiological, psychological, and social factors might contribute to the development of depression disorder. Publications suggest that depression has a relationship with pesticide exposure and metabolic disorders [4,5].

The purpose of pesticides is to poison and destroy insects, but they are not selectively toxic to the target organism and therefore toxic to other species, including human beings [6]. Organophosphate insecticides (OPIs) have been generally utilized all over the world [7], instead of organochlorine pesticides, accounting for 30% of the global insecticide market demand [8]. In 2001, OPIs accounted for 70% of total pesticides; however, the ratio has fallen after then and kept within 30 and 40%, based on the statistical data of the US Environmental Protection Agency (EPA) [9]. It has been obvious that prolonged low-dose exposure to OPIs probably exerts an adverse influence on numerous human body systems, such as the brain, respiratory system, nervous system, reproductive system, and endocrine system [10,11,12,13]. Although OPIs have been gradually restricted in the US since the Food Quality Protection Act passed in 1996, the extensive consumption of OPIs is easily accessible to a large number of people via multiple sources [8]. In addition, the detection of OPI metabolites in urine samples from the general population indirectly supports the former assumption [7]. Organophosphates are primarily metabolized in the liver and excreted through the urinary tract [6]. The organophosphates’ metabolic process produces nonspecific metabolites known as dialkyl phosphate, including dimethyl phosphate (DMP), diethyl phosphate (DEP), dimethyl thiophosphate (DMTP), diethyl thiophosphate (DETP), dimethyl dithiophosphate (DMDTP), and diethyl dithiophosphate (DEDTP), which could be detected in urine and used as exposure biomarkers for organophosphates.

Previous studies have shown that there is a link between OPIs and depression risk in US adults [11]; however, associations between OPIs and depression score as well as depression severity remain unclear. More evidence is needed to prove these viewpoints. Following the methods of Hongwei Cai et al. [14], the authors’ purpose was to figure out whether depression score and severity have associations with dialkyl phosphate metabolites in urine. Therefore, this paper focuses on not only depression score but also depression severity, using a large sample size and including several age groups.

## 2. Methods

### 2.1. Study Design

The data stem from the National Health and Nutrition Examination Survey (NHANES) 2015–2016 and 2017–2018 cycles. The investigation was permitted by The National Center for Health Statistics (NCHS) Research Ethics Review Board, and written informed consent was acquired. More information is available on the website (https://wwwn.cdc.gov/nchs/nhanes/Default.aspx (accessed on 30 September 2024)).

**Scheme 1:**
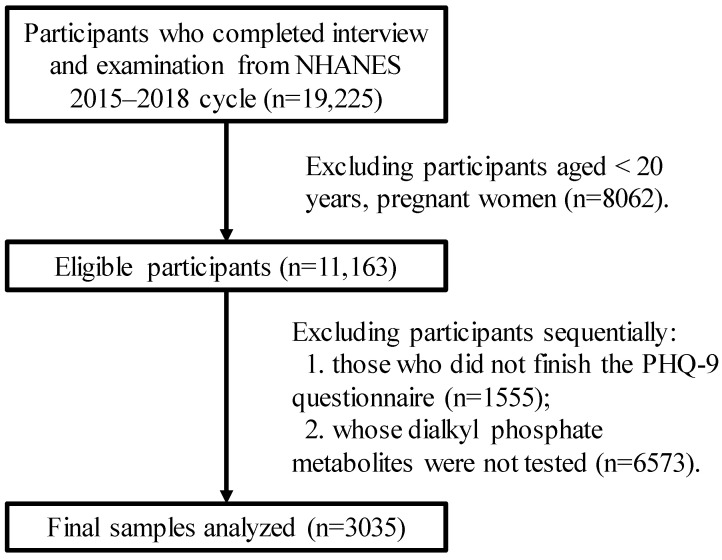
Flowchart of eligible participants included in this study.

This study used data on population, physical examination, factors of daily life, and urine laboratory examinations. Solid phase extraction coupled with isotope dilution-ultrahigh performance liquid chromatography-tandem mass spectrometry was used to detect urine specimens. The lower limit of detection for dialkyl phosphate metabolites was 0.1 ng/mL and the highest found concentration was 622.000 ng/mL. Some covariates defined were as follows: (a) active physical activity status was at least 150 min per week of moderate intensity aerobic exercise and/or 75 min per week of energetic aerobic exercise [15], and (b) smoking status “yes” contained former and current smokers, while “no” stood for “never”. All kinds of dialkyl phosphate metabolites in urine were adjusted by creatinine. Eventually, 3035 individuals were involved in the succeeding analysis based on the exclusion standards (Scheme 1).

### 2.2. Measurement of Depression Score

The Patient Health Questionnaire-9 (PHQ-9) is a nine-item depression screening tool used to determine the frequency of depression symptoms in the past two weeks. Scores range from 0 to 3, and every question has four different choices. Therefore, the total points, ranging from scores of 0 to 27, could be used to determine whether a participant suffers from depression disorder or not. A PHQ-9 score of 0–4 represents a participant who does not have depression, 5–9 a participant with mild depression, 10–14 moderate depression, 15–19 moderately severe depression, and 20–27 severe depression, respectively [16]. A PHQ-9 score ≥ 10 has a good performance in diagnosing major depression, with 88% sensitivity and specificity [14].

### 2.3. Statistics

All the analyses considered the complex design factors and sampling weights. Numerical variables are presented as weighted means and standard deviations (SDs). Categorical variables are presented as weighted proportions. Chi-square and Kruskal–Wallis H tests were conducted to compare categorical or numerical variables by degrees of depression, respectively. Linear regression was conducted to discover the associations of depression score with five types of dialkyl phosphate metabolites. Multiple logistic regression was conducted to discover the associations of depression severity with these metabolites. Three models were gradually conducted in order to adjust for possible confounding effects from various combinations of covariates. Age and gender were adjusted in Model 1. Race/ethnicity, education background, and family poverty–income ratio (PIR) were further adjusted in Model 2. Covariates in Model 2 including Body Mass Index (BMI), waist circumference, smoking status, and active physical activity were further adjusted in Model 3. The depression scores and depression severity were treated as the numerical variable and categorical variable, respectively. All the analyses were employed in IBM SPSS (version 22.0). Two-sided *p* < 0.05 was considered statistically significant.

## 3. Results

Table 1 summarizes the demographic characteristics. For total participants, the weighted mean age was 48 years. 64.1% of the people with moderate-to-severe depression (i.e., major depression with a PHQ-9 score ≥ 10) are female. The proportion of white people with minimal, mild, and moderate-to-severe depression was 64.5%, 65.2%, and 55.2%. People with a college degree or above accounted for 66.2%, 56.1%, and 52.3% of populations with minimal, mild, and moderate-to-severe depression. The proportion of rich individuals (i.e., PIR > 3) decreased with depression severity, but the proportion of poor people (PIR ≤ 1) increased with depression severity. In addition, depression severity was associated with BMI category, waist circumference, smoking status, and physical activity (*p* < 0.001).

Table 2 summarizes the overall prevalence of mild and major depression among various subpopulations in the US. The prevalence of both mild (11.6%, 95% CI: 10.4–12.8%) and major (6.5%, 95% CI: 5.5–7.4%) depression among women was higher than men’s (8.5%, 95% CI: 7.4–9.6%; 3.9%, 95% CI: 3.1–4.7%), and especially young and middle-aged women (i.e., age < 60 years) suffered badly (4.4%, 95% CI: 3.5–5.2%). Non-Hispanic white people were most vulnerable to depressive disorder, having a prevalence of 8.0% (95% CI: 6.9–9.0%) for mild depression and 3.4% (95% CI: 2.7–4.2%) for major depression. An increased susceptibility to depression was observed in populations and was linked with obesity, smoking status, and physical activity.

Table 3 summarizes the results of multiple logistic regression and linear regression of depression score and severity on DMP, DEP, DMTP, DETP, and DMDTP. Firstly, in the fully adjusted Model 3, the continuous PHQ-9 score had associations with a higher presence of DMP in urine (odds ratio (OR) = 1.77; 95% CI, 1.05–2.98; *p* = 0.032), and the estimation of effect size was not influenced by the various combinations of covariates. It also showed that individuals suffering from mild depression had a 13% increase (95% CI: 12–13%) in the odds of exposure to DMP compared to subjects with minimal depression after the PHQ-9 score was further categorized. Secondly, the significance remained with more covariates added in Model 2 and Model 3. When considering minimal depression as a control group, the effect size of major depression with the existence of DMP still had statistical significance in Model 3 (OR = 2.75; 95% CI: 2.74–2.76; *p* < 0.001). A PHQ-9 score of 5 or more was strongly associated with increased concentrations of DMP (OR = 5.56; 95% CI: 5.53–5.59; *p* < 0.001), DEP (OR = 2.58; 95% CI: 2.56–2.59; *p* < 0.001), DMTP (OR = 2.81; 95% CI: 2.80–2.82; *p* < 0.001), DETP (OR = 1.20; 95% CI: 1.19–1.21; *p* < 0.001), and DMDTP (OR = 2.41; 95% CI: 2.39–2.42; *p* < 0.001). Multiple logistic regression Model 2 revealed that major depression was linked to increased concentrations of DMP (OR = 1.47; 95% CI: 1.47–1.47; *p* < 0.001), DEP (OR = 1.31; 95% CI: 1.31–1.31; *p* < 0.001), DMTP (OR = 1.16; 95% CI: 1.16–1.16; *p* < 0.001), and DMDTP (OR = 1.09; 95% CI: 1.09–1.10; *p* < 0.001). Table 4 is a description of characteristics of study subjects according to dialkyl phosphate metabolites.

## 4. Discussion

A nationally representative sample of US adults was examined in this cross-sectional study, revealing a positive correlation between depression scores measured by the PHQ-9 and the presence of dialkyl phosphate metabolites in urine, regardless of other established risk factors. Our findings demonstrate that the influence of dialkyl phosphate metabolites on depression severity, particularly among those who suffer from major depression, has statistical significance after being adjusted by confounding covariates. Furthermore, analysis of the depression score suggests that these metabolites might contribute to the risk of developing major depression.

A survey conducted by the Food and Agriculture Organization of the United Nations (FAO) has revealed that countries such as the United States and China engage in notable agricultural activities and are the world’s largest consumers of pesticides. Consequently, these countries’ governments have expressed significant concern regarding the biomonitoring of environmentally exposed populations [6]. Additionally, those developed countries that possess the financial and technological capabilities to conduct epidemiological studies and population biomonitoring on a large scale are also actively involved. Some of these countries conduct population surveys or extensive cohort studies to measure urinary dialkyl phosphates metabolites, enabling the monitoring of organophosphate exposure. The NHANES has been carried out since 1999 in the US. In recent years, urinary dialkyl phosphates have emerged as a common method of assessing exposure to organophosphates in epidemiological investigations. However, the detection of urinary dialkyl phosphates does not provide specificity with respect to the organophospate from which they were derived, or their toxicological potency [17]. This is a cross-sectional study that explores the correlation between depression score as well as severity and urinary dialkyl phosphate concentrations in the implementation of pesticides in agriculture.

Based on our knowledge, this marks the first study to explore the relationship between depression scores and dialkyl phosphate metabolites in urine among the general population. Prior research conducted by Jennifer et al. revealed that aircraft maintenance workers exposed to organophosphates exhibited a heightened likelihood of reporting depression symptoms. Notably, the incidence of depression was significantly associated with varying levels of self-reported exposure, ranging from low (OR = 1.21) to moderate (OR = 1.68) to high (OR = 2.70), regardless of the exposure route, whether it was through contact (OR = 1.68), inhalation (OR = 2.52), or ingestion (OR = 2.55) [18]. Similarly, Virginia et al. observed that UK sheep farmers with a history of low-level exposure to organophosphates were more prone to experiencing symptoms of anxiety and depression compared to unexposed controls, even after accounting for demographic and psychosocial risk factors [19]. However, the sample size of these prior studies was smaller than the current investigation, and they did not assess the concentrations of dialkyl phosphate metabolites in urine. In our current research, we have corroborated an independent impact of dialkyl phosphate metabolites on the risk of depression. Unlike other studies primarily focusing on organophosphate exposure, we have also presented evidence suggesting a potential association between dialkyl phosphate metabolites and mild depression, underscoring the importance of early intervention in depression management.

There may be underlying mechanisms that connect dialkyl phosphate metabolites to depression. Previous studies have employed repeated low-dose exposure to the organophosphate agent diisopropyl fluorophosphate (DFP) on rats over a five-day period, followed by assessments at three months post-exposure. These experiments observed symptoms of chronic depression in the exposed rats. Notably, chronic low-dose DFP exposure was associated with hippocampal neuronal damage, evident through Fluoro-Jade staining [20]. In another experiment, rats exposed to DFP exhibited significant long-term impairments in mood, anxiety, depression, and aggressive traits. Brain section analysis revealed significant losses of neuronal nuclei antigen principal neurons, parvalbumin inhibitory interneurons, and neurogenesis. Additionally, there was an increase in astrogliosis, microglial neuroinflammation, and mossy fiber sprouting [21]. There is abundant evidence in vivo demonstrating that organophosphate exposure leads to a reduction in N-acetylaspartate (NAA), a marker of neuronal metabolism, which has been confirmed to be reduced also in humans suffering from depression in recent articles and meta-analyses [22,23]. Hence, the observed association between urinary dialkyl phosphate metabolites and depression severity may be underpinned by physiopathological mechanisms involving neuronal metabolism. This overlap in findings suggests that organophosphate-induced reduction of NAA could contribute to the neurobiological basis of depression observed in exposed populations. This potential mechanism aligns with the chronic neuronal damage and neuroinflammation observed in animal models of organophosphate exposure. By disrupting neuronal metabolism and integrity, organophosphates may predispose individuals to depressive symptoms or exacerbate existing vulnerabilities, providing a plausible physiopathological explanation for the associations identified in the study.

The strengths of this study are as follows. Firstly, it is a groundbreaking attempt to assess the relationship between depression scores and urinary dialkyl phosphate metabolites. This exploration fills a crucial gap in our understanding of the potential environmental factors that may contribute to depressive symptoms. Secondly, the study boasts a large and nationally representative sample of the US general population. This ensures that the estimates of depression prevalence are not only accurate but also applicable to a wide range of adults in the United States, regardless of their race or ethnic background. The generality of these findings is a crucial aspect, as it enables a more comprehensive understanding of the issue at a national level. Moreover, the study avoids the limitations of the commonly used binary categorization of the PHQ-9 score (i.e., major depression or not). Instead, it incorporates the score into the models in a more nuanced and flexible manner. This approach allows for the detection of associations between urinary dialkyl phosphate metabolites and depression across a broader spectrum, including mild depression, which is often overlooked in traditional binary classifications.

Despite the strengths of this study, there are indeed several limitations that should be addressed. Firstly, the depression score was evaluated using a self-administered questionnaire, which may introduce subjectivity and potential biases. Although the questionnaire is widely used and validated, it is important to note that the externally validated depression status was not available for comparison or validation purposes [14]. This limits the accuracy and reliability of the depression score reported in this study. Secondly, the study focused on the associations between depression score and five types of organophosphates metabolites. However, DEDTP was excluded from the analysis due to concentrations that were too low to detect. This exclusion may have limited the scope of the study and prevented the examination of potential associations between DEDTP and depression. Finally, the cross-sectional nature of the survey design poses challenges in making causal inferences. The association found in this study might be affected by other unmeasured factors, and the causal relationship remains unclear. To strengthen the evidence and establish more robust causal relationships, prospective cohort studies or Mendelian randomization studies are needed. These studies can provide a longitudinal perspective and allow for the examination of temporal relationships between exposures and outcomes, while also accounting for potential confounders.

In conclusion, our findings indicate a positive and independent association between urinary dialkyl phosphate metabolites and depression score as well as severity among the general population in the United States. Given these findings, limiting the use of organophosphate pesticides and implementing suitable screening for the concentrations of these metabolites in urine may contribute to preventing the onset or progression of depression. Due to the study design, it is not known whether the observed association is specific to depression symptoms or whether it is common to other psychiatric disorders. The use of Mendelian randomization or longitudinal data would provide stronger evidence.

## Figures and Tables

**Table 1:** General characteristics of study participants in the NHANES 2015–2018 cycle according to depression severity.

Characteristics	Total (*n* = 3035)	Minimal (*n* = 2276)	Mild (*n* = 510)	Moderate to Severe (*n* = 249)	*p*
Demographic					
Age (year)	48 ± 17	48 ± 17	47 ± 18	46 ± 17	<0.001
20–39, %	35.9	34.7	38.3	42.5	
40–59, %	35.3	35.6	35.0	32.6	
≥60, %	28.8	29.7	26.7	24.9	
Gender, female (%)	50.1	47.1	57.4	64.1	<0.001
Race/ethnicity (%)					<0.001
Mexican American	8.7	8.6	9.0	9.1	
Other Hispanic	6.3	5.9	6.9	9.4	
Non-Hispanic White	63.9	64.5	65.2	55.2	
Non-Hispanic Black	11.7	11.5	11.8	14.3	
Other race	9.3	9.6	7.1	12.0	
Education (%)					<0.001
Less than high school	11.7	10.2	15.7	17.7	
High school or equivalent	24.8	23.5	28.2	30.0	
College or above	63.6	66.2	56.1	52.3	
PIR (%)					<0.001
≤1	13.2	10.3	20.0	28.2	
1–3	35.8	33.8	41.3	43.7	
>3	51.0	55.8	38.7	28.1	
Anthropometry					
BMI (%)					<0.001
Thin	5.3	30.1	6.4	7.9	
Normal	24.3	25.3	20.7	22.0	
Overweight	30.7	32.8	24.9	22.2	
Obese	39.7	37.1	48.0	48.0	
Waist circumference (cm)	100.2 ± 17.2	99.6 ± 16.8	102.3 ± 18.6	101.7 ± 17.4	<0.001
Behaviors					
Smoking (%)	43.6	37.6	50.8	68.2	<0.001
Active physical activity (%)	48.6	48.4	49.9	48.4	<0.001

Note: data are presented as weighted means with standard deviation (SD) for continuous variables and weighted proportions for categorical variables. Abbreviations: PIR, ratio of family income to poverty; BMI, body mass index.

**Table 2:** Prevalence of depression among various subpopulations in the US.

Characteristics	Mild (95% CI, %)	Moderate-to-Severe (95% CI, %)
Gender		
Male	8.5 (7.4–9.6)	3.9 (3.1–4.7)
Female	11.6 (10.4–12.8)	6.5 (5.5–7.4)
Age by gender		
<60, male	5.7 (4.8–6.6)	2.3 (1.7–2.9)
≥60, male	3.1 (2.4–3.9)	1.7 (1.2–2.2)
<60, female	8.1 (7.0–9.2)	4.4 (3.5–5.2)
≥60, female	4.2 (3.4–5.0)	2.3 (1.7–2.9)
PIR		
≤1	5.4 (4.5–6.3)	3.0 (2.3–3.7)
1–3	8.9 (7.7–10.0)	4.2 (3.4–5.1)
>3	4.8 (4.0–5.7)	1.7 (1.2–2.3)
Race/ethnicity		
Mexican American	3.5 (2.7–4.2)	1.9 (1.3–2.4)
Other Hispanic	2.4 (1.7–3.0)	1.5 (1.0–2.0)
Non-Hispanic White	8.0 (6.9–9.0)	3.4 (2.7–4.2)
Non-Hispanic Black	4.8 (4.0–5.7)	2.7 (2.0–3.3)
Other race	2.6 (1.9–3.2)	1.3 (0.8–1.7)
BMI		
Thin	1.2 (0.7–1.6)	0.7 (0.4–1.0)
Normal	4.1 (3.3–4.9)	1.9 (1.3–2.5)
Overweight	5.6 (4.7–6.6)	2.3 (1.7–2.9)
Obese	9.8 (8.7–11.0)	5.4 (4.5–6.3)
Smoking	5.5 (4.6–6.4)	3.6 (2.8–4.3)
Active physical activity	9.1 (8.0–10.2)	4.6 (3.7–5.4)

Note: data are presented as ratios with 95% CIs. Abbreviations: PIR, ratio of family income to poverty; BMI, body mass index.

**Table 3:** Associations between depression score and urinary dialkyl phosphate metabolites.

	Model 1		Model 2		Model 3	
	OR (95% CI)	*p*	OR (95% CI)	*p*	OR (95% CI)	*p*
DMP						
PHQ-9 score	1.19 (0.71–2.00)	0.507	1.47 (0.87–2.47)	0.148	1.77 (1.05–2.98)	0.032
PHQ-9 category						
≤4	Ref.		Ref.		Ref.	
5–9	0.87 (0.87–0.87)	<0.001	0.95 (0.95–0.95)	<0.001	1.13 (1.12–1.13)	<0.001
≥10	1.25 (1.25–1.26)	<0.001	1.47 (1.47–1.47)	<0.001	2.75 (2.74–2.76)	<0.001
PHQ-9 score ≥ 5	3.86 (3.84–3.88)	<0.001	5.04 (5.01–5.07)	<0.001	5.56 (5.53–5.59)	<0.001
PHQ-9 score ≥ 10	1.30 (1.28–1.31)	<0.001	1.23 (1.22–1.24)	<0.001	0.94 (0.93–0.95)	<0.001
DEP						
PHQ-9 score	0.81 (0.44–1.49)	0.498	0.93 (0.51–1.70)	0.809	1.09 (0.60–2.00)	0.773
PHQ-9 category						
≤4	Ref.		Ref.		Ref.	
5–9	0.70 (0.70–0.70)	<0.001	0.78 (0.77–0.78)	<0.001	0.78 (0.78–0.79)	<0.001
≥10	1.24 (1.24–1.24)	<0.001	1.31 (1.31–1.31)	<0.001	1.88 (1.87–1.88)	<0.001
PHQ-9 score ≥ 5	2.26 (2.24–2.27)	<0.001	2.19 (2.17–2.20)	<0.001	2.58 (2.56–2.59)	<0.001
PHQ-9 score ≥ 10	0.36 (0.35–0.36)	<0.001	0.37 (0.36–0.37)	<0.001	0.39 (0.39–0.40)	<0.001
DMTP						
PHQ-9 score	1.06 (0.70–1.61)	0.790	1.23 (0.80–1.87)	0.346	1.37 (0.90–2.09)	0.144
PHQ-9 category						
≤4	Ref.		Ref.		Ref.	
5–9	0.84 (0.84–0.84)	<0.001	0.88 (0.88–0.89)	<0.001	0.94 (0.94–0.94)	<0.001
≥10	1.00 (1.00–1.00)	0.306	1.16 (1.16–1.16)	<0.001	1.83 (1.82–1.83)	<0.001
PHQ-9 score ≥ 5	2.32 (2.31–2.33)	<0.001	2.83 (2.82–2.84)	<0.001	2.81 (2.80–2.82)	<0.001
PHQ-9 score ≥ 10	0.71 (0.70–0.71)	<0.001	0.66 (0.66–0.66)	<0.001	0.57 (0.57–0.58)	<0.001
DETP						
PHQ-9 score	0.62 (0.37–1.04)	0.068	0.67 (0.40–1.12)	0.122	0.81 (0.48–1.36)	0.422
PHQ-9 category						
≤4	Ref.		Ref.		Ref.	
5–9	0.64 (0.64–0.64)	<0.001	0.70 (0.70–0.70)	<0.001	0.67 (0.67–0.67)	<0.001
≥10	0.78 (0.78–0.78)	<0.001	0.80 (0.80–0.80)	<0.001	0.88 (0.87–0.88)	<0.001
PHQ-9 score ≥ 5	1.09 (1.09–1.10)	<0.001	1.09 (1.09–1.10)	<0.001	1.20 (1.19–1.21)	<0.001
PHQ-9 score ≥ 10	0.24 (0.24–0.24)	<0.001	0.29 (0.29–0.30)	<0.001	0.36 (0.35–0.36)	<0.001
DMDTP						
PHQ-9 score	0.86 (0.51–1.44)	0.565	0.94 (0.56–1.58)	0.802	1.11 (0.66–1.88)	0.693
PHQ-9 category						
≤4	Ref.		Ref.		Ref.	
5–9	0.77 (0.76–0.77)	<0.001	0.77 (0.77–0.77)	<0.001	1.00 (1.00–1.01)	<0.001
≥10	0.92 (0.92–0.92)	<0.001	1.09 (1.09–1.10)	<0.001	1.76 (1.76–1.77)	<0.001
PHQ-9 score ≥ 5	1.79 (1.78–1.80)	<0.001	2.33 (2.32–2.35)	<0.001	2.41 (2.39–2.42)	<0.001
PHQ-9 score ≥ 10	0.57 (0.57–0.58)	<0.001	0.39 (0.39–0.39)	<0.001	0.39 (0.39–0.40)	<0.001

Note: Model 1 was adjusted for age and gender. Model 2 was further adjusted for race/ethnicity, educational level, and ratio of family income to poverty. Model 3 included covariates in Model 2 plus body mass index, waist circumference, smoking status, and physical activity. Abbreviations: DMP, dimethyl phosphate; DEP, diethyl phosphate; DMTP, dimethyl thiophosphate; DETP, diethyl thiophosphate; DMDTP, dimethyl dithiophosphate.

**Table 4:** General characteristics of study participants in the NHANES 2015–2018 cycle according to dialkyl phosphate metabolites.

Characteristics	DMP	*p*	DEP	*p*	DMTP	*p*	DETP	*p*	DMDTP	*p*
Demographic										
Age (year)		<0.001		<0.001		<0.001		<0.001		<0.001
20–39	−5.961 (−6.265, −5.644)		−5.746 (−6.042, −5.432)		−6.275 (−6.639, −5.813)		−6.836 (−7.159, −6.491)		−6.940 (−7.204, −6.590)	
40–59	−5.905 (−6.204, −5.566)		−5.699 (−6.008, −5.412)		−6.220 (−6.562, −5.793)		−6.778 (−7.087, −6.427)		−6.911 (−7.168, −6.528)	
≥60	−5.818 (−6.085, −5.520)		−5.604 (−5.908, −5.351)		−6.059 (−6.410, −5.678)		−6.699 (−6.987, −6.328)		−6.820 (−7.080, −6.442)	
Gender		<0.001		<0.001		<0.001		<0.001		<0.001
Male	−5.969 (−6.265, −5.666)		−5.770 (−6.068, −5.466)		−6.274 (−6.607, −5.789)		−6.850 (−7.133, −6.480)		−6.946 (−7.204, −6.600)	
Female	−5.825 (−6.110, −5.529)		−5.614 (−5.921, −5.320)		−6.118 (−6.487, −5.735)		−6.723 (−7.030, −6.374)		−6.832 (−7.109, −6.470)	
Race/ethnicity		<0.001		<0.001		<0.001		<0.001		<0.001
Mexican American	−5.791 (−6.045, −5.485)		−5.737 (−6.035, −5.465)		−6.135 (−6.457, −5.723)		−6.746 (−7.062, −6.420)		−6.906 (−7.179, −6.599)	
Other Hispanic	−5.904 (−6.175, −5.589)		−5.699 (−6.016, −5.431)		−6.192 (−6.533, −5.702)		−6.784 (−7.050, −6.353)		−6.918 (−7.178, −6.535)	
Non-Hispanic White	−5.889 (−6.173, −5.598)		−5.662 (−5.977, −5.366)		−6.166 (−6.528, −5.754)		−6.784 (−7.079, −6.410)		−6.862 (−7.116, −6.479)	
Non-Hispanic Black	−5.985 (−6.277, −5.665)		−5.740 (−6.028, −5.466)		−6.370 (−6.742, −5.968)		−6.895 (−7.200, −6.504)		−7.063 (−7.303, −6.746)	
Other race	−5.845 (−6.198, −5.556)		−5.699 (−6.005, −5.405)		−6.249 (−6.596, −5.773)		−6.757 (−7.043, −6.331)		−6.850 (−7.137, −6.519)	
Education		<0.001		<0.001		<0.001		<0.001		<0.001
Less than high school	−5.851 (−6.204, −5.535)		−5.783 (−6.060, −5.491)		−6.222 (−6.533, −5.726)		−6.827 (−7.075, −6.401)		−6.962 (−7.228, −6.541)	
High school or equivalent	−5.958 (−6.277, −5.693)		−5.768 (−6.071, −5.450)		−6.291 (−6.619, −5.866)		−6.845 (−7.142, −6.484)		−6.929 (−7.180, −6.606)	
College or above	−5.865 (−6.152, −5.571)		−5.644 (−5.950, −5.365)		−6.148 (−6.537, −5.735)		−6.744 (−7.072, −6.383)		−6.862 (−7.133, −6.493)	
PIR		<0.001		<0.001		<0.001		<0.001		<0.001
≤1	−5.926 (−6.220, −5.593)		−5.770 (−6.078, −5.490)		−6.277 (−6.651, −5.869)		−6.832 (−7.127, −6.478)		−6.983 (−7.228, −6.627)	
1–3	−5.924 (−6.250, −5.623)		−5.715 (−6.028, −5.424)		−6.254 (−6.625, −5.814)		−6.854 (−7.146, −6.484)		−6.929 (−7.184, −6.555)	
>3	−5.840 (−6.132, −5.565)		−5.654 (−5.946, −5.363)		−6.132 (−6.488, −5.703)		−6.728 (−7.039, −6.380)		−6.834 (−7.109, −6.473)	
Anthropometry										
BMI		<0.001		<0.001		<0.001		<0.001		<0.001
Thin	−5.889 (−6.175, −5.521)		−5.619 (−5.989, −5.264)		−6.265 (−6.570, −5.600)		−6.707 (−7.064, −6.382)		−6.832 (−7.114, −6.456)	
Normal	−5.848 (−6.155, −5.570)		−5.633 (−5.937, −5.381)		−6.104 (−6.454, −5.720)		−6.642 (−6.940, −6.306)		−6.804 (−7.069, −6.456)	
Overweight	−5.850 (−6.136, −5.557)		−5.693 (−5.968, −5.408)		−6.173 (−6.531, −5.751)		−6.724 (−7.058, −6.391)		−6.868 (−7.137, −6.467)	
Obese	−5.957 (−6.281, −5.641)		−5.733 (−6.047, −5.418)		−6.297 (−6.648, −5.858)		−6.914 (−7.163, −6.543)		−6.963 (−7.216, −6.658)	
Behaviors										
Smoking		<0.001		<0.001		<0.001		<0.001		<0.001
Yes	−5.993 (−6.316, −5.771)		−5.824 (−6.151, −5.507)		−6.362 (−6.748, −5.943)		−6.865 (−7.184, −6.587)		−6.994 (−7.265, −6.707)	
No	−5.862 (−6.153, −5.568)		−5.688 (−5.961, −5.353)		−6.183 (−6.493, −5.732)		−6.770 (−7.094, −6.359)		−6.879 (−7.137, −6.493)	
Active physical activity		<0.001		<0.001		<0.001		<0.001		<0.001
Yes	−5.907 (−6.181, −5.623)		−5.708 (−6.031, −5.413)		−6.241 (−6.582, −5.799)		−6.833 (−7.142, −6.450)		−6.915 (−7.184, −6.558)	
No	−5.866 (−6.169, −5.553)		−5.672 (−5.967, −5.374)		−6.150 (−6.529, −5.727)		−6.748 (−7.041, −6.400)		−6.875 (−7.137, −6.512)	

Notes: all values of metabolites were corrected for creatinine and adjusted by log10, and presented as weighted median with first and third quartiles. Abbreviations: PIR, ratio of family income to poverty; BMI, body mass index; DMP, dimethyl phosphate; DEP, diethyl phosphate; DMTP, dimethyl thiophosphate; DETP, diethyl thiophosphate; DMDTP, dimethyl dithiophosphate.

## Data Availability

The data stemmed from the combination of the National Health and Nutrition Examination Survey (NHANES) 2015–2016 and 2017–2018 cycles. More information cis available on the website (https://wwwn.cdc.gov/nchs/nhanes/Default.aspx, accessed on 30 September 2024).
